# Dysbiosis of the Gut Microbiome Is Associated With Histopathology of Lung Cancer

**DOI:** 10.3389/fmicb.2022.918823

**Published:** 2022-06-14

**Authors:** Xiong Qin, Ling Bi, Wenxiao Yang, Yiyun He, Yifeng Gu, Yong Yang, Yabin Gong, Yichao Wang, Xiaoxia Yan, Ling Xu, Haibo Xiao, Lijing Jiao

**Affiliations:** ^1^Department of Thoracic Surgery, Shanghai Pulmonary Hospital, Tongji University, Shanghai, China; ^2^Department of Oncology, Yueyang Hospital of Integrated Traditional Chinese and Western Medicine, Shanghai University of Traditional Chinese Medicine, Shanghai, China; ^3^Department of Cardiothoracic Surgery, Xinhua Hospital, School of Medicine, Shanghai Jiao Tong University, Shanghai, China; ^4^Yueyang Hospital of Integrated Traditional Chinese and Western Medicine, Institute of Clinical Immunology, Shanghai University of Traditional Chinese Medicine, Shanghai, China

**Keywords:** 16S rRNA sequencing, lung cancer, gut microbiome, biomarkers, histopathology

## Abstract

Lung cancer is a malignancy with high incidence and mortality worldwide. Previous studies have shown that the gut microbiome plays an important role in the development and progression of metabolic cancers. However, data on the characteristics of the gut microbiome with different histopathology types of lung cancer remain scant. We collected stool samples from 28 healthy people (HP) and 61 lung cancer patients. The lung cancer patients were classified into three types according to their histopathology: Atypical Adenomatous Hyperplasia/Adenocarcinoma *in situ* (AAH/AIS), Minimally Invasive Adenocarcinoma (MIA), and Invasive Adenocarcinoma (IA). In addition, we employed 16S rRNA gene amplicon sequencing to analyze the characteristics of the gut microbiome in these patients. Our analysis revealed that the categorized cancer patients had unique intestinal flora characteristics, and had lower density and flora diversity compared to healthy people. Besides, the structure of the flora families and genera was more complex, and each group presented specific pathogenic microbiota. The patients in the AAH/AIS group and HP group had relatively similar flora structure compared with the IA and MIA groups. In addition, we identified several flora markers that showed significant changes with the development of lung cancer. Lung cancer gut microbiota showed a decrease in short-chain fatty acids (SCFAs) producing and anti-inflammatory bacteria compared to healthy people, while some pathogenic bacteria such as proinflammatory or tumor-promoting bacteria were more abundant in lung cancer patients. On the other hand, the Kyoto Encyclopedia of Genes and Genomes (KEGG) and Clusters of Orthologous Group (COG) annotation demonstrated suppression of some dominant metabolism-related pathways in lung cancer. These findings provide new biomarkers for the diagnosis and prognostic assessment of lung cancer and lay the basis for novel targeted therapeutic strategies for the prevention and treatment of lung cancer.

**Clinical Trial Registration:**

[www.ClinicalTrials.gov], identifier [NCT03244605].

## Introduction

Lung cancer is one of the most aggressive and prevalent types of malignancy that leads to high morbidity and mortality (Allemani et al., 2018). Over 80% of lung cancer incidences are non-small cell lung cancer (NSCLC) (Wagner et al., 2020), which include adenocarcinoma (AC) and squamous cell carcinoma (SCC). However, with the development of individualized and targeted therapy for lung cancer, traditional pathological classification no longer meets the treatment requirements. It is, therefore, important to characterize the lung cancer subtypes based on the existing diagnostic criteria, coupled with more sensitive and specific diagnostic and prognostic markers.

Intestinal bacteria is a systemic metabolic product, which mediates disease resistance through metabolism, immunity, inflammation, and other mechanisms. Few studies have evaluated the interplay between the microbiome and lung cancer. Recent studies have also shown that intestinal flora has a unique population, which expresses in different cancers such as lung, breast, pancreatic, brain, and bone cancers (Nejman et al., 2020). In the treatment of lung cancer, intestinal flora can improve the efficacy and sensitivity of chemotherapy, radiotherapy, or immunotherapy, and reduce treatment-related toxicities (Cheng et al., 2020). In addition to carcinogenic effects, intestinal flora can also inhibit the development of cancer (Kadosh et al., 2020). The intestinal flora modulated cancer development by regulating its microenvironment, the host’s immune system, as well as other metabolites (Finlay et al., 2020). Thus, the gut microbiome could correlate with the development of lung cancer, but evidence for the interplay between the microbiome and lung cancer is insufficient and cannot yet be used to predict tumor progression and prognosis.

An ideal diagnostic or prognostic index should have high specificity and sensitivity. Novel indexes such as intestinal flora have received considerable prospects for clinical application. To define biomarkers in the development of early lung adenocarcinoma, we explored the role played by intestinal flora changes using 16S rRNA sequencing and then attempted to correlate the intestinal flora changes with the development of infiltrating carcinoma. These data provided a theoretical basis for the accurate diagnosis and classification of early lung cancer.

## Materials and Methods

### Samples

The 89 fecal samples for 16S rRNA sequencing were obtained from 28 healthy people and 61 lung cancer patients initially diagnosed by histopathology and computed tomography (CT). The lung cancer patients were further divided into 3 groups based on different histopathology as prescribed by WHO classification on Tumors of the Lung, Pleura, Thymus, and Heart in 2015, which include Atypical Adenomatous Hyperplasia/Adenocarcinoma *in situ* patients (AAH/AIS group, *n* = 8), minimally invasive adenocarcinoma patients (MIA group, *n* = 18), invasive adenocarcinoma patients (IA group, *n* = 35). None of the patients received therapy, such as chemotherapy, radiation therapy, targeted therapy, immunotherapy, or surgery before sample collection. We excluded patients who had one of the following conditions: congestive cardiac failure, respiratory failure, renal failure, severe liver dysfunction, consumption of probiotics or antibiotics within 1 month before admission. The control group was of 28 healthy people (HP group) who did not use any type of antibiotics or probiotics within 1 month before admission. Fresh fecal samples from all the participants were collected by the fecal sample collection kit (MGI Tech Co., Ltd., China) for intestinal microbial gene testing. The fecal samples were transferred into a sterilized tube containing stabilizer N-octylpyridine, which is a reliable reagent suitable for storage and transportation at room temperature. Then the fecal samples were frozen at –80°C immediately until DNA extraction. This study was conducted by the Declaration of Helsinki. The study was approved by the ethics committee of Yueyang Hospital of Integrated Traditional Chinese and Western Medicine Affiliated with Shanghai University of Traditional Chinese Medicine (NO.2016-059). Each patient gave signed informed consent before the study. The clinical trial registration date was August 9, 2017, and the registry number was NCT03244605.

### Fecal DNA Extraction and 16S Sequencing

Microbial DNA was extracted from 89 fecal samples (61 fecal samples from lung cancer patients and 28 fecal samples from healthy people) by QIAamp^®^ Fast DNA Stool Mini Kit following the manufacturer’s protocol. Briefly, the V3–V4 variable regions of the bacterial 16S rRNA gene were amplified by polymerase chain reaction (PCR) using universal primers 338F: (ACTCCTACGGGAGGCAGCAG) 806R:GGACTACHVGGGTWTCTAAT). The extracted DNA was purified by silica gel and then quantified using a Quantus™ Fluorometer. The PCR cycle conditions included an initial denaturation at 95°C for 3 min; followed by 30 cycles at 95°C for 30 s, primer annealing at 52°C for 30 s, and extension at 72°C for 45 s; followed by a final elongation at 72°C for 10 min. The PCR products were then analyzed in 2% agarose gel. Subsequently, purified amplicons were pooled in equimolar amounts, and paired-end sequenced on Illumina HiSeq/MiniSeq for genome analysis.

### Microbiome Data Analysis

The raw FASTQ files were first de-multiplexed, quality-filtered using chimera check, and then merged using FLASH (Magoč and Salzberg, 2011) with the sequences which were processed using the Cutadapt v1.3 and QIIME v1.8.0 (Cock et al., 2010). Briefly, forward, and reverse bacterial 16S rRNA reads were merged with a minimum length of 200 bps, and then we used the pick_open_reference method in the QIIME analysis to perform OTU clustering. The clustering algorithm used Uclust, and the database used the Greengenes 2013-08 release^1^ version, and the similarity threshold was 80% for all sequences. Thereafter, we performed Operational Taxonomic Units (OTUs) division and statistical analysis, and the remaining parameters were the default parameters for QIIME. The index of observed species, Chao, Shannon, Sobs and Simpson were used to calculate alpha (α) diversity metrics. The beta (β) diversity measurements including Principal Component Analysis (PCA) and Principal Coordinates Analysis (PCoA) were used by the unweighted UniFrac metric. The PCA and PCoA were based on unweighted uniFrac distance. The statistical significance was evaluated using analysis of similarities (ANOSIM). In addition, the Linear Discriminant Analysis (LDA) Effect Size (LEfSe) method was used to evaluate the influence of each differentially abundant taxon. We further conducted an correlation network analysis to identify the co-occurring intestinal microbes under different histopathology types. To analyze the correlation network, we calculated the Spearman correlation between different groups of phylum using the R package cooccur. Subsequently, significant and robust correlations (*P*-value < 0.01, |ρ| ≥ 0.6) were used to construct a network using the R package psych. Gephi (v0.9) was then used to construct network figures. Finally, pathway enrichment analysis was performed using the Kyoto Encyclopedia of Genes and Genomes (KEGG) and the Phylogenetic Investigation of Communities by Reconstruction of Unobserved States (PICRUSt) 2.0 database (Kanehisa et al., 2008; Langille et al., 2013; Douglas et al., 2020).

### Statistical Analyses

Statistical tests were performed in R (3.0.2; R Foundation for Statistical Computing) and Prism software (Graph Prism7.0 Software Inc., CA, United States). Data were expressed as a mean ± standard deviation (SD) and the differences among the groups were evaluated by Wilcoxon rank-sum test. The Wilcoxon rank-sum test (for two groups) or Kruskal-Wallis test (for more than two groups) was used to analyze the diversity between multi-groups. Besides, Fisher’s exact test was performed on categorical variables, whiles the chi-square test was used for categorical variables. A value *P* < 0.05 was considered statistically significant.

## Results

### Patient Characteristics

Clinical characteristics of all the participants were listed in Table 1. No difference was observed in age, sex, disease stage, smoking status and family history (*P* > 0.05).

**TABLE 1:** Baseline characteristics of health people and non-small cell lung cancer (NSCLC) patients.

Characteristics	Total (*n* = 89)	HP (*n* = 28)	AAH/AIS (*n* = 8)	MIA (*n* = 18)	IA (*n* = 35)	*P-value*
Age, years Mean ± *SD*	55.88 ± 10.87	58.79 ± 11.16	49.00 ± 5.61	53.67 ± 12.34	56.26 ± 10.17	0.110
**Sex, *n* (%)**						
Male	30 (33.71)	11 (39.29)	2 (25.00)	7 (38.89)	10 (28.57)	0.731
Female	59 (66.29)	17 (60.71)	6 (75.00)	11 (61.11)	25 (71.43)	
**Smoking status, *n* (%)**						
Smoker	10 (11.24)	5 (17.86)	0 (0.00)	3 (16.67)	2 (5.71)	0.279
Non-smoker	79 (88.76)	23 (82.14)	8 (100.00)	15 (83.33)	33 (94.29)	
**Family history, *n* (%)**						
Yes	7 (7.87)	0 (0.00)	0 (0.00)	1 (5.56)	6 (17.14)	0.098
No	82 (92.13)	28 (100.00)	8 (100.00)	17 (94.44)	29 (82.86)	
**Disease stage, *n* (%)**						
IA	–	–	0 (0.00)	17 (94.44)	30 (85.71)	
IB	–	–	0 (0.00)	1 (5.56)	3 (8.57)	0.529
IIA	–	–	0 (0.00)	0 (0.00)	0 (0.00)	
IIB	–	–	0 (0.00)	0 (0.00)	2 (5.71)	
**EGFR mutation, *n* (%)**						
L858R	–	–	0 (0.00)	1 (5.56)	8 (22.86)	
19-del	–	–	0 (0.00)	0 (0.00)	4 (11.43)	0.074
Unknown	–	–	8 (100.00)	17 (94.44)	23 (65.71)	
**Solitary/multiple nodule, *n* (%)**						
Solitary	–	–	2 (25.00)	8 (44.44)	18 (51.43)	0.396
Multiple	–	–	6 (75.00)	10 (55.56)	17 (48.57)	
**Defecation, *n* (%)**						
Normal	62 (69.66)	28 (100.00)	7 (87.50)	15 (83.33)	27 (77.14)	0.067
Abnormal	27 (30.34)	0 (0.00)	1 (12.50)	3 (16.67)	8 (22.86)	

*HP, healthy people; AAH, atypical adenomatous hyperplasia; AIS, adenocarcinoma in situ; MIA, minimally invasive adenocarcinoma; IA, invasive adenocarcinoma.*

### Clustering Analysis of Operational Taxonomic Unit

**FIGURE 1:**
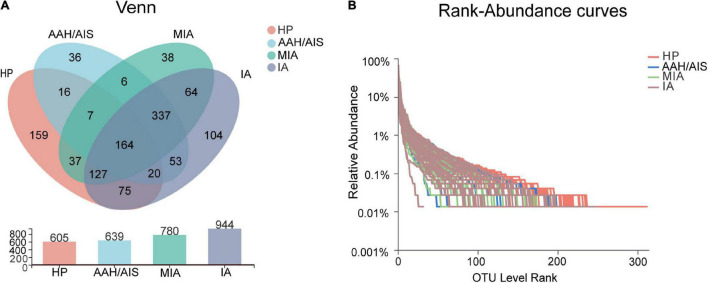
Taxonomic analysis of 16S rRNA sequence data. **(A)** Venn diagram of OTU shared among the four groups. **(B)** Rank-Abundance curves of intestinal flora in four groups of samples.

A total of 1,243 Operational Taxonomic Units (OTUs) were annotated for subsequent analysis, including 15 phyla, 81 families, 253 genera, and 555 species of gut microbes (Figure 1A). The coverage of 16S rRNA sequencing was 400–440 bp and the average length of these fragments was 415 bp (Supplementary Figure 1A). The data showed that the sobs index tended to be stable as sampling increased, which indicated that the depth of our sample sequencing met the analysis requirements for the diversity of intestinal flora (Supplementary Figure 1B).

### Taxonomic Analysis of the 16S rRNA Sequence Data

To explore the features of the gut microbial community of the lung cancer patients, the relative microbiota taxon abundance in the lung cancer groups was compared with healthy people. The predominant genera were defined as those comprising greater than 1% of the total gut bacteria. Bacterial taxonomy distribution of the three lung cancer groups demonstrated increased density and clustering compared to the healthy controls group. In addition, a total of 605 OTUs were obtained for the HP group, 639 OTUs for the AAH/AIS group, 780 OTUs for the MIA group, and 944 OTUs for the IA group as shown by the Venn diagrams (Figure 1A). The number of unique OTUs in each group was 36, 38, 104, and 159 in AAH/AIS, MIA, IA, and HP groups, respectively. In addition, the HP and the lung cancer groups had a total of 446 shared OTUs, indicating that there was the high similarity between the structure of the intestinal flora of the healthy group and the lung cancer patients (Figure 1A). Rank-Abundance curves showed that the intestinal flora of the healthy group had higher abundance and diversity compared to the lung cancer groups (Figure 1B).

### The Alpha Diversity of the Gut Microbiota

**FIGURE 2:**
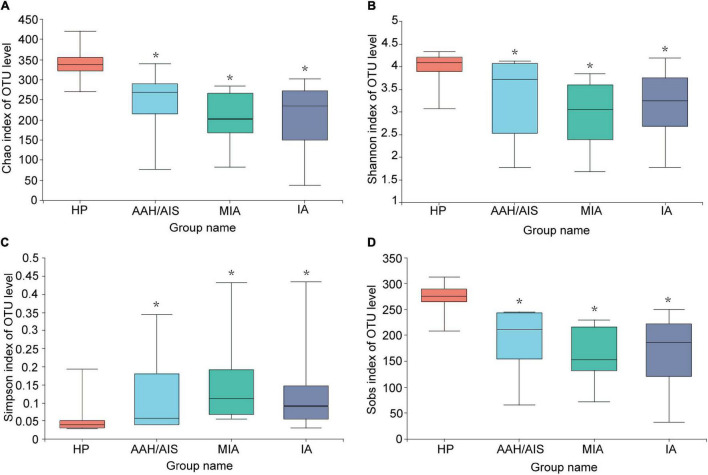
Comparison of Alpha diversity index of intestinal flora among the four groups. **(A)** Microbial alpha diversity of Chao index. **(B)** Shannon index. **(C)** Simpson index. **(D)** Sobs index. *Indicates *P* < 0.05 compared to the HP group.

To investigate the diversity of the bacterial species in the gut ecosystem in each group, the microbial alpha diversity was measured as shown in Figure 2. Alpha diversity evaluates the diversity of microbial communities in a region, reflecting the richness and evenness. We obtained data such as species abundance by observation of various index values such as Chao, Shannon, Sobs, and Simpson index. Community richness can be measured by Chao index, while community diversity indices includes Shannon index and Simpson index. Sobs index represents the number of species observed in the sample (OTU number). The Chao, Shannon, Sobs index are positively correlated with the richness and diversity while the Simpson index is negatively correlated with them. We then employed a *t*-test to define the significance of the differences in the index values between the four groups. The Chao, Shannon, or Sobs index (*P*< 0.05) demonstrated that the diversity index of the HP group was significantly higher compared to the three lung cancer groups, while the Simpson index was lower compared to the three lung cancer groups (*P*< 0.05). Our results demonstrated that the intestinal flora of lung cancer patients was significantly different from in the HP group, and the gut microbiota abundance and diversity of the lung cancer patients were lower than the HP group. In addition, there was no significant differences in the indices of the different lung cancer groups (*P* > 0.05).

### The Beta Diversity Analysis of the Gut Microbiota

**FIGURE 3:**
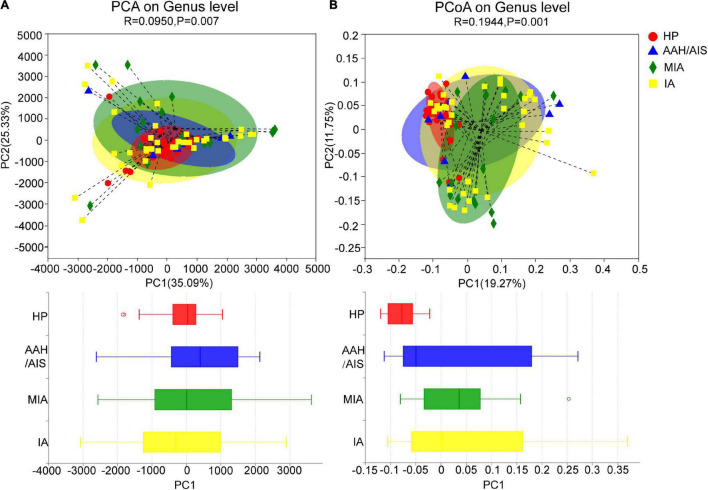
Beta diversity analysis of intestinal flora. **(A)** PCA analysis of intestinal flora in four groups of samples. **(B)** PCoA analysis of intestinal flora in four groups of samples.

The Beta (β) diversity was used to evaluate the similarities and differences of between-group diversity of each group, including principal component analysis (PCA) and principal coordinates analysis (PCoA) based on unweighted UniFrac distance. The more similar the community composition of the samples is, the closer they are to each other in the PCA or PCoA diagram. Therefore, samples with high similarity in community structure tend to cluster together, while those with very different communities are far apart. We performed the PCA analysis between the four groups as shown in Figure 3A. When PC1 (35.09%) and PC2 (25.33%) were taken as the abscissa and ordinate, respectively, the four groups were well distinguished (*P* = 0.007), demonstrating that the four groups had significant differences in the composition of the intestinal bacteria. Besides, in the PCoA analysis (Figure 3B), when PC1 (19.27%) and PC2 (11.75%) were taken as the abscissa and ordinate, respectively, the four groups were farther apart in the coordinate chart (*P* = 0.001), which indicated that there was a significant difference in species diversity between the four groups.

In summary, our data showed that there were significant differences in the species diversity and community composition of the intestinal flora between the lung cancer patients and healthy controls, as well as certain differences in the diversity and structure of the intestinal flora between the three different pathological subgroups of lung cancer. However, the results of Beta diversity can only illustrate the general similarities and differences of diversity between each group. Therefore, the clear information on detailed differences between the four groups were further reflected by subsequent species taxonomic profiling at different levels of biological classification.

### Variation Analysis

#### Species Specificity in Multi-Level Tests

**FIGURE 4:**
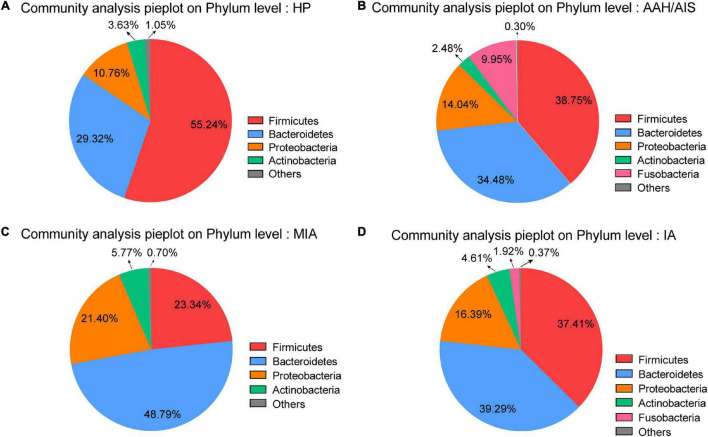
Major OTUs at Phylum level in the HP group vs. the three lung cancer subgroups. **(A)** Major OTUs at Phylum level in the HP group. **(B)** Major OTUs at Phylum level in the AAH/AIS group. **(C)** Major OTUs at Phylum level in the MIA group. **(D)** Major OTUs at Phylum level in the IA group.

At the phylum level, Firmicutes, Bacteroidetes, and Proteobacteria were the most common phyla identified in the three lung cancer groups, contributing 87.27% (AAH/AIS), 93.53% (MIA), and 93.09% (IA) of the gut bacteria, respectively. Firmicutes, Bacteroidetes, Proteobacteria, and Acidobacteria contributed to 98.95% of the gut bacteria in the HP group (Figure 4A). The lung cancer groups especially the MIA group had a significantly lower abundance of Firmicutes, a relatively higher abundance of Proteobacteria, Bacteroidetes, and Fusobacteria compared to the HP group. On the other hand, the AAH/AIS group showed a relatively low abundance of Acidobacteria (Figures 4B–D). The ratio of Firmicutes to Bacteroidetes can reflect the homeostasis of intestinal flora. The Firmicutes/Bacteroidetes ratio in the HP group was 1.88, while in the lung cancer group, the ratio was 1.12 (AAH/AIS), 0.48 (MIA), and 0.95 (IA), respectively.

**FIGURE 5:**
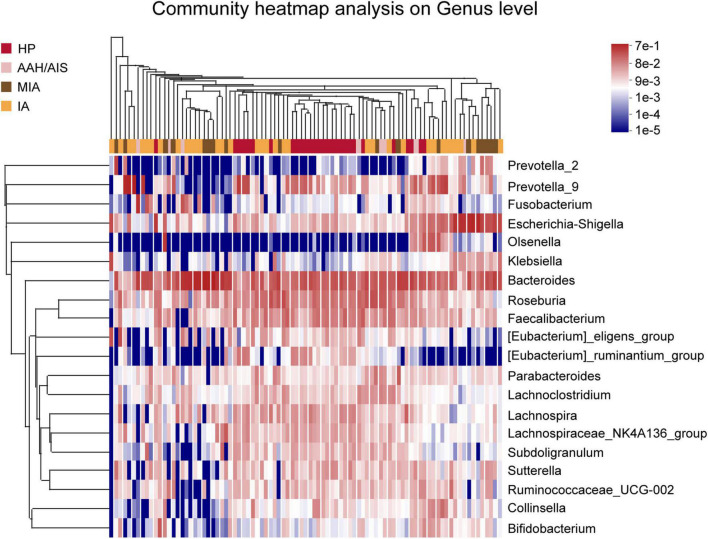
Species classification heat map analysis. The color gradient from blue to red indicates that the distance between the samples is from near toward far.

In addition, analysis of relative abundance showed a clear difference between the taxa with high and low abundance were distinguished, and the color gradient were used to reflect the similarity and difference of the composition of multiple samples at each classification level. As shown in Figure 5, the difference between the four groups of samples can be seen intuitively according to the change in the color gradient.

#### Gut Microbial Signature in Lung Cancer Patients

**FIGURE 6:**
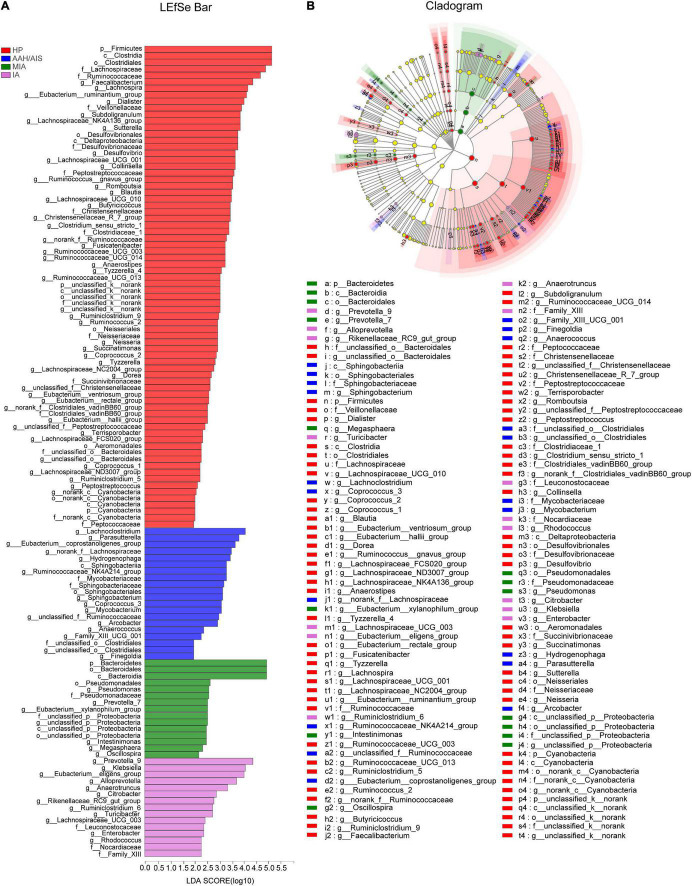
Identification of gut microbiota composition and abundance across the four groups. **(A)** Histogram of the distribution of LDA values for LEfSe analysis of intestinal flora in four groups of samples. **(B)** Evolutionary map of species branching for LefSe analysis of intestinal flora in four groups of samples.

The multi-level LEfSe was used to analyze biomarkers between the lung cancer patients with different histopathology and the healthy controls. Our results showed that dominant fecal gut microbiota was specific to the histopathological types of lung cancer. There were 74, 20, 15, and 15 bacterial taxonomic clades that were significantly different in HP, AAH/AIS, MIA, IA groups, respectively [log10 (LDA score) > 2] (Figure 6A).

We also analyzed the evolutionary relatedness of the intestinal flora species as shown in Figure 6B. The data showed that the species were divergent, which was in sync with the LDA value distribution data. The data showed that the dominant flora in each group of lung cancer patients was significantly different from the healthy people, and there were also significant differences in the characteristic flora in the lung cancer patients based on the different pathological types.

**FIGURE 7:**
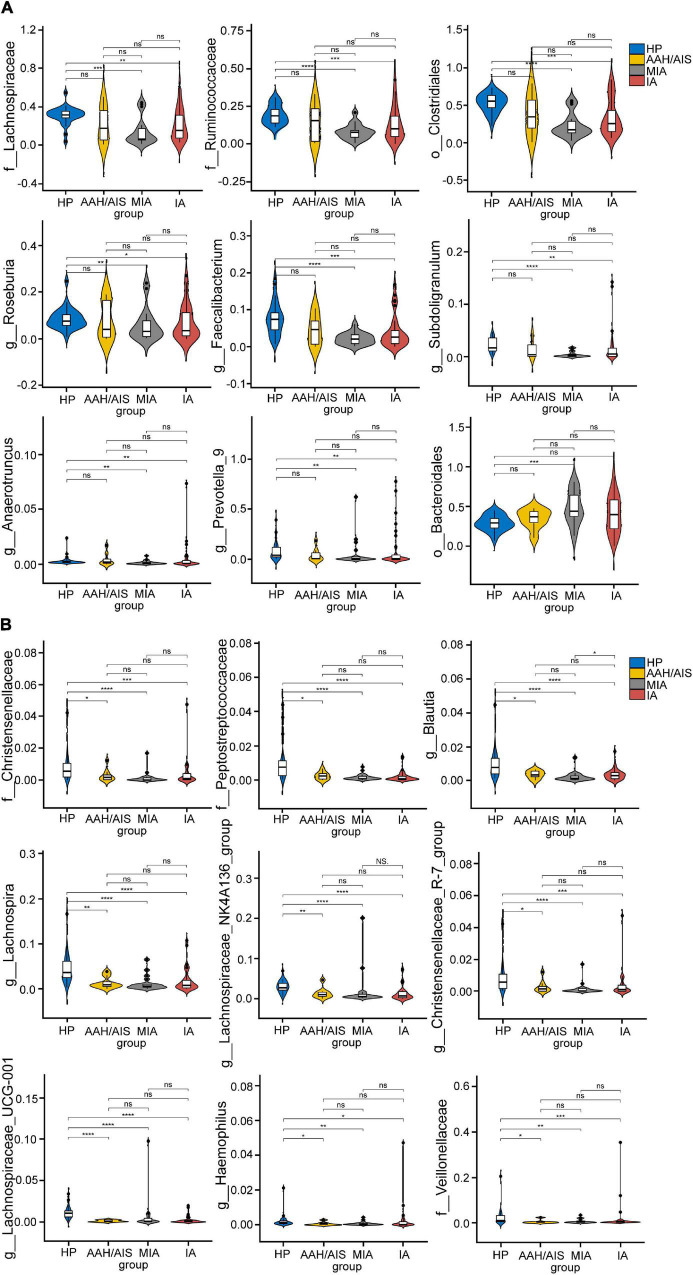
The comparison of relative abundant microbiome between each group. **(A)** The Characteristic flora which are significantly HP group is significantly different from MIA, IA, and no difference. **(B)** The Characteristic flora which are significantly different between HP and the other three group. *P< 0.05, **P< 0.01, ***P< 0.001, ****P< 0.0001, ns, no significance.

The flora evolution analysis showed the relative content of these dominant bacteria (Figure 7). In the HP group, p_Firmicutes, c_Clostridia, and o_Clostridiales were shown to be the most significant, while in AAH/AIS group, g_Lachnoclostridium, g_Parasutterella, and g_Eubacterium_coprostanoligenes had the highest abundance. On the other hand, in the MIA group, p_Bacteroidetes, o_Bacteroidale, and c_Bacteroidia were shown to be the most significant genus, while in the IA group, g_Prevotella_9, g_Klebsiella, and g_Eubacterium_eligens were most represented. Besides, in the HP group, the dominant bacteria group was classified at a high level, while the different lung cancer groups were significantly reflected in the low-level classification. Further analysis showed that o_Bacteroidales, o_Clostridiales, f_Lachnospiraceae, f_Ruminococcaceae, g_Anaerotruncus, g_Faecalibacterium, g_Prevotella_9, g_Roseburia, and g_Subdoligranulum in HP group was significantly different from MIA, IA, but not with AAH/AIS group (Figure 7A). On the other hand, f__Peptostreptococcaceae, f_Christensenellaceae, f_Veillonellaceae, g_Blautia, g_Christensenellaceae_R-7_group, g_Haemophilus, g_Lachnospira, g_Lachnospiraceae_NK4A136_group, and g_Lachnospiraceae_UCG-001 were significantly different from the other three groups (Figure 7B). These florae features may be related to the development of lung cancer.

Moreover, several specific genera were presented in both lung cancer patients and healthy people. According to the LEfSe analysis, the genera of Lachnospiraceae, Ruminococcaceae, and Eubacterium were predominantly identified in both cancer patients and healthy people. Specifically speaking, the genera of Lachnospiraceae were in both healthy people and IA group. The genera of Ruminococcaceae were both enriched in healthy people and AAH/AIS group. Eubacterium genera were simultaneously identified in healthy people and three lung cancer subgroups AAH/AIS, MIA, and IA group.

**FIGURE 8:**
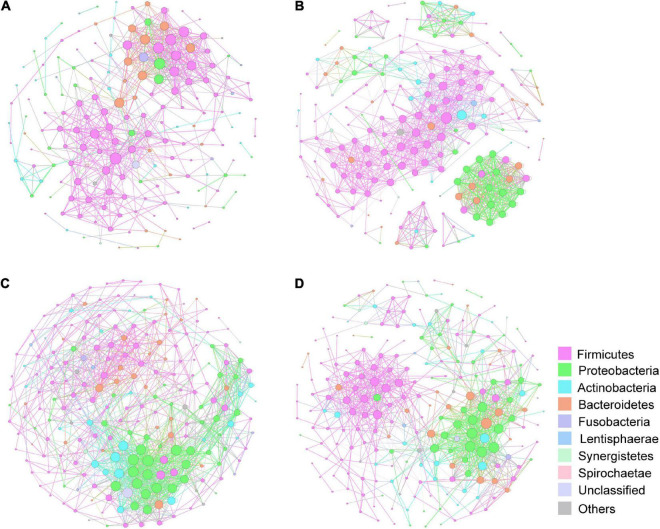
Correlation network of the gut microbiome in the four groups. The correlation coefficient was calculated with Spearman rank correlation test (| ρ| ≥ 0.6). Gephi (v0.9) was used for network construction. **(A)** Correlation networks in HP. **(B)** Correlation networks in AAH/AIS. **(C)** Correlation networks in MIA. **(D)** Correlation networks in IA. Each circle represents the average relative abundance of a microbial species in that state. Node sizes are scaled according to their degrees of connections.

Constructed networks revealed that samples from the HP had fewer edges, a lower average degree and lower nodes than those from the lung cancer group, which indicated that there were fewer significant correlations of phylum (Supplementary Table 1). In AAH/AIS group, average weighted degree, density and clustering coefficient were higher than the other three groups, demonstrating a elevation in the network complexity. Co-occurrence was also found among species of the Proteobacteria in AAH/AIS, MIA, IA environments (Figures 8B–D), however, such co-occurrence was missing in the healthy environment (Figure 8A).

#### Functional Profile of the Gut Microbiome in Non-small Cell Lung Cancer

The KEGG and COG pathway analyses were performed to explore potential differences in the functions of the microbiome in lung cancer patients vs. healthy individuals.

**FIGURE 9:**
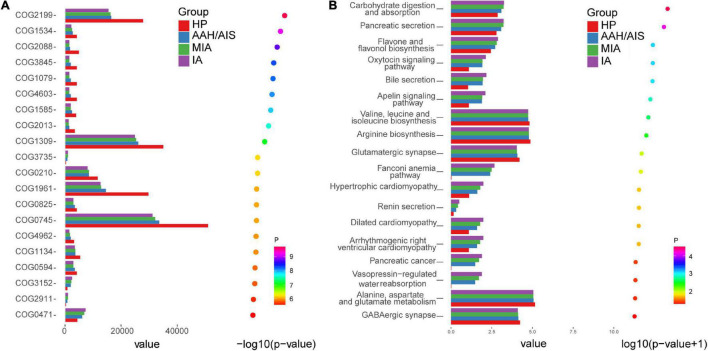
COG pathway and KEGG analysis in the four groups. **(A)** The relative abundance of COG pathway differentially enriched in the four groups. **(B)** The relative abundance of KEGG pathway differentially enriched in the four groups.

Although the functional analyses showed significant similarity between the lung cancer patients and the control group, the microbiome of the lung cancer patients was abundant in pathways such as carbohydrate digestion and absorption, which was proportional to the development of lung cancer. On the other hand, the KEGG analysis showed clustering of valine, leucine, and isoleucine biosynthesis, arginine biosynthesis, and glutamatergic synapse, which showed lower abundance in the lung cancer patients than the healthy controls (Figure 9A). In addition, diguanylate cyclase (COG2199) and RNA-binding protein (COG1534) of the ABC (ATP-binding cassette) transporter system were significantly downregulated in lung cancer patients compared to the healthy controls, which might be promoting utilization of glucose or ribose/galactoside to regulate energy. In addition, exported protein (COG2911) ortholog was upregulated in the lung cancer patients compared to the healthy controls (*P* < 0.05) (Figure 9B).

## Discussion

Adenocarcinoma is the most common type of pathology in NSCLC. With the development of imaging techniques like High Resolution Computed Tomography (HRCT), CT imaging, Positron Emission Tomography-Computed Tomography (PET-CT), and Magnetic Resonance Imaging (MRI), the detection rate of early lung adenocarcinoma has significantly improved. However, how to analyze the prognosis of patients with early lung adenocarcinoma is particularly critical.

There are three major types of pathology in early lung adenocarcinoma, including adenocarcinoma *in situ* (AIS), minimally invasive adenocarcinoma (MIA), and invasive adenocarcinoma (IA) with a maximum tumor diameter of ≤ 3 cm (Travis et al., 2015). Besides the AIS, there is atypical adenomatous hyperplasia (AAH) with very similar morphology. Although the CT imaging of the above lesions is mainly in the form of ground-glass nodules, their prognosis is quite different. AAH can be observed and followed up without surgery for years; AIS and MIA could be treated by lobectomy without regional lymph node dissection, with a 5-year survival rate of 100%; while submerged IA with predominantly appendicular growth requires lobectomy and regional lymph node dissection, with a 5-year survival rate of 67% (Detterbeck et al., 2017). With the increase in the detection rate of pulmonary ground-glass nodules, it is essential to classify the degree of malignancy of the nodules. Unfortunately, the ground glass nodules have similarities and overlap in histomorphology, which blocks accurate diagnosis and treatment. Presently, experienced pathologists identified the types of early lung adenocarcinoma based on infiltrating carcinoma components in the lesion, but there are no specific biological markers of infiltrating carcinoma components in lesions, especially for the early lung adenocarcinoma patients. Therefore, it is urgent to explore non-invasive and economical screening modalities which could easily detect samples with high positive rates.

Recent studies have shown that intestinal flora can be used in the diagnosis of human diseases such as tumors (Zheng et al., 2020; Leng et al., 2021). Intestinal flora is a large group of microorganisms that colonize the intestines, and their homeostasis plays an important role in regulating the development of human diseases and is referred to as the “second genome” (Qin et al., 2010) or “a new organ” (Donaldson et al., 2016). Previous data has demonstrated a pathogenic association between the microorganisms and the gut-lung axis (Gut-lung axis) (Budden et al., 2017), which is the basis for the regulation of lung cancer by the intestinal flora microenvironment. The intestines and the lung regulate each other through the gut-lung axis, which relies on various biological structures such as embryonic homology, mucosal immune channels, and neurological channels. Besides, the intestinal microenvironment could influence the occurrence, development, treatment, and prognosis of lung cancer through various pathways. In our study, we showed that the lung cancer group had significant differences from the healthy group, which is consistent with previous reports (Liu et al., 2019a). Thus, the microbiota has high sensitivity in early lung adenocarcinoma compared to blood tumor markers such as carcinoembryonic antigen (CEA), carbohydrate antigen 125 (CA125), and squamous cell carcinoma (SCC) antigen.

The α- and β-diversity results of lung cancer patients with different histopathology types did not show any significant differences, but the HP and AAH/AIS groups showed high similarity, while the IA group was similar to the MIA group. Together, these differences were not statistically significant but was confirmed by specific flora structure.

Moreover, at the phylum level, Firmicutes were significantly higher in the HP group compared to the AAH/AIS, MIA, and IA groups, while the ratio of Firmicutes to Bacteroidetes was lower than in the HP group. Previous data demonstrated that all butyrate-producing bacteria belong to the Firmicutes. Besides, butyrate is one of the most important fatty acids associated with anti-inflammatory activity, cell proliferation, induction of regulatory T cell differentiation, and apoptosis through activation of signaling pathways (O’Keefe, 2016; Feng et al., 2018). High rates of Firmicutes/Bacteroidetes phylum are frequently observed in healthy adults, as previously demonstrated using a large gut microbiome cohort study (Zhong et al., 2019). Reduced Firmicutes/Bacteroidetes ratio has been shown to be associated with dysbiosis of gastrointestinal tract metabolism, which results in low concentration of circulating short-chain fatty acids, and then influenced elements for host systemic immunity and systemic inflammation (Liu et al., 2019b). This data shows that there is a disrupted balance of gut microbiota in lung cancer patients and the presence of distinct microbiota profiles from those of precancerous lesions.

The characterizations in family and genus levels were more complex and significantly varied from each group, presenting a more diverse pathogenic population. Our results showed that the Lachnospiraceae and Blautia genera were suppressed in lung cancer patients, which was in agreement with previous studies (Liu et al., 2019a; Zhang et al., 2019). The Lachnospiraceae genera of the Clostridium family belongs to Firmicutes phylum, which was suppressed in each lung cancer group compared to the HP. Lachnospiraceae can protect the host against cancer by producing butyric acid which plays an important role in the suppression of tumor growth, regulation of immunity, and participation in anti-inflammatory reactions (Daniel et al., 2017). Each lung cancer group exhibited a decreased abundance of the Blautia genus belonging to the Firmicutes phylum, which has a role in digesting complex carbohydrates. The suppression of the Blautia genus was also seen in irritable bowel syndrome, non-alcoholic fatty liver diseases, Crohn’s disease, and diabetes. However, the specific roles of these common specific florae and their importance need further confirmation (Zhang et al., 2019). Our results indicated that the composition and development of bacterial communities varied in lung cancer with a different course. Therefore, it is feasible to speculate that some microbiome might be used for diagnosis, prognosis, therapeutics or fecal microbiota transplantation in lung cancer.

Our data also showed that there was a lower abundance of Faecalibacterium, Prevotella, Roseburia, and Subdoligranulum, Anaerotruncus genera in lung cancer patients in IA and MIA groups compared with HP, but no difference with AAH/AIS group. Faecalibacterium was reported as a “favorable” gut microbiome, which can enhance systemic and anti-tumor immune responses mediated by increased antigen presentation, and improved effector T cell functions as well as the tumor microenvironment, which modulates the response of melanoma patients to anti-programmed death-1(PD-1) immunotherapy (Gopalakrishnan et al., 2018). It was also shown that patients on Cytotoxic T Lymphocyte-associated Antigen-4 (CTLA-4) blockade with a higher abundance of Faecalibacterium had a prolonged PFS compared to those with a higher abundance of Bacteroidales in the gut microbiome (Chaput et al., 2017). Thus, these findings demonstrated that Faecalibacterium plays an important role in immunotherapy. Prevotella belongs to the Prevotaceae family of Bacteroides. It has a diverse bacterial species and is a dominant genus in the human intestine. It is negatively associated with metabolic diseases such as obesity and diabetes (Lukens et al., 2014). In our study, we showed that Prevotella decreased with lung cancer progression. Roseburia genus has been shown to produce short-chain fatty acids, especially butyric acid, which affects colon movement with anti-inflammatory properties and has the potential of being a probiotic (Sanders et al., 2019). Studies have shown that the occurrence of colorectal cancer may be related to the reduction of the Roseburia (Bisht et al., 2021). Our findings showed that the reduction of Roseburia genus was associated with the occurrence and progression of lung cancer.

In the healthy people group, the majority of gut bacteria were associated with the production of short-chain fatty acids (SCFAs), the regulation of the immune system, and the modulation of metabolism. The microbial genera in healthy people were characterized by a higher abundance of beneficial bacteria that promote the restoration of gut microenvironment balance, and some of them were identified as the next-generation probiotics (Singh and Natraj, 2021). However, these beneficial gut microbiota were not significantly observed in either of the three subgroups of lung cancer patients. On the contrary, most of the beneficial gut microbiota were significantly decreased in lung cancer patients, and some pathogenic bacteria such as proinflammatory or tumor-promoting bacteria were more abundant in lung cancer patients. Lachnoclostridium (Liang et al., 2020), Pseudomonas (Rathje et al., 2020), Eubacterium_xylanophilum_group (Zhang et al., 2020), Megasphaera (Lee et al., 2016), Klebsiella (Jian et al., 2020), Citrobacter (Mullineaux-Sanders et al., 2019), and Enterobacter (Yurdakul et al., 2015) were regarded as pathogenic bacteria involved in inducing inflammation or generating cancer development. Further studies will be conducted to investigate the mechanisms of how these gut microbiota influence lung cancer occurrence, progression and prognosis.

Gut microbiota interaction is a key factor of the microbial equilibrium. Our correlation networks results demonstrated that the microbial network was complexed in the early stage of lung cancer. These results suggest changes in gut microbial homeostasis in the early stages of lung cancer. Our results also showed the network indices including network density, clustering coefficient and average degree was significantly different between HP and lung cancer. Whether they could be used as quantitative parameters to assess cancer risk and homeostasis of the lung microbiome requires further study.

In addition, the predicted 16S functions showed that there were significant differences between the different groups. These results were in agreement with our hypothesis which showed that in the early stages of lung carcinogenesis, there was no significant disease progression in the AAH/AIS group compared with the HP group. Therefore, the structure of the intestinal flora was closer compared to that of healthy individuals. In contrast, patients in the IA and MIA groups were at a later stage of lung cancer development and had a more altered flora structure compared to the healthy individuals. We thought that the tumor cells may produce metabolites and exhibit different characteristics, and the metabolic disorders and tumor abnormalities may progressively worsen as the disease progresses. On the other hand, the harmful flora in the lung cancer group was also reduced. To a certain extent, this was also a manifestation of the imbalance of the intestinal flora. The Anaerotruncus genus belongs to the Clostridium and participates in the carbohydrate metabolism pathway. The final metabolites are beneficial acetic and butyric acids. A previous study demonstrated that the abundance of Anaerotruncus was significantly increased in the intestinal flora of a mouse model with non-alcoholic fatty liver-related cancer fed on high diet cholesterol (Zhang et al., 2021). Besides, Anaerotruncus was significantly enriched in the uterine microbiome of patients with endometrial cancer (Walther-António et al., 2016).

The KEGG and COG analysis also showed significant differences in the intestinal flora between lung cancer patients and healthy individuals. Further functional analysis of the intestinal bacteria revealed that the flora in lung cancer patients was associated with carbohydrate digestion and absorption. Our findings showed the same metabolic disorders and tumor abnormalities in the intestinal flora. These bacteria may shed different microbial bioactive molecules and affect the utilization of valine, leucine and isoleucine biosynthesis, arginine biosynthesis, glutamate synthesis, glucose, ribose/galactoside by the host. Firmicutes could alter undigested carbohydrates and proteins into acetate, which then produces energy for the organism (Liu et al., 2019a). Furthermore, the reduced abundance of the ABC (ATP-binding cassette) transporter system suggested the potential for energetic and metabolic alterations in the microbiota in lung cancer. This observation is consistent with the hypothesis that lung cancer is fundamentally a metabolic disease and that lung cancer patients often exhibit coexisting metabolic disorder phenotypes and pathologies.

Existing data focused on comparative analysis of intestinal flora changes, which investigated the characteristics of the changes in intestinal flora in different lung cancer histopathology. However, to our knowledge, there are no studies on the relationship between intestinal flora and the development of different histopathological lung cancers. Our study compared the structure of intestinal flora in healthy individuals and patients with different histopathology types in early stage lung cancer. These findings may provide new insights into the development of lung cancer, suggesting that the intestinal flora may be closely related to the progression of lung cancer which can help determine the stage of the disease. Using various bioinformatics methods, such as α-diversity and β-diversity analysis, we identified intestinal flora in lung cancer patients. The population structure of the lung cancer patients was different from the healthy population, which was consistent with previous results. However, there was no overall imbalance in the structure of the intestinal flora in patients with early lung cancer, indicating that the imbalance does not significantly affect the occurrence and development of lung cancer. Meanwhile, the observation of dynamic observation with larger scale were needed in the future.

## Conclusion

We classified lung cancer patients with different histopathology types and performed a detailed study to characterize the structure of intestinal flora. Our results revealed that the different histopathology types of lung cancer were associated with structural changes in the intestinal flora. AAH/AIS group had a more similar structure to the HP group, while the IA and MIA groups showed a greater change in the colony structure. Lung cancer gut microbiome showed a decrease in SCFA-producing and anti-inflammatory bacteria compared to healthy people, while some pathogenic bacteria such as proinflammatory or tumor-promoting bacteria were more abundant in lung cancer patients. Our findings would provide clues for the use of intestinal flora as a biomarker in the assessment of lung cancer progression and the effective development of targeted therapy.

## Data Availability Statement

The datasets presented in this study can be found in online repositories. The names of the repository/repositories and accession number(s) can be found below: https://www.ncbi.nlm.nih.gov/, PRJNA772805.

## Ethics Statement

The studies involving human participants were reviewed and approved by the Ethics Committee of Yueyang Hospital of Integrated Traditional Chinese and Western Medicine Affiliated to Shanghai University of Traditional Chinese Medicine. The patients/participants provided their written informed consent to participate in this study.

## Author Contributions

XQ, HX, and LJ contributed to the conception and design of the study. LB, WY, YH, and XY performed the experiments. YGu, YY, and YW performed the statistical analysis. WY, LB, and LJ wrote the first draft of the manuscript. LX and YGo edited the manuscript. All authors contributed to manuscript revision, read, and approved the submitted version.

## Conflict of Interest

The authors declare that the research was conducted in the absence of any commercial or financial relationships that could be construed as a potential conflict of interest.

## Publisher’s Note

All claims expressed in this article are solely those of the authors and do not necessarily represent those of their affiliated organizations, or those of the publisher, the editors and the reviewers. Any product that may be evaluated in this article, or claim that may be made by its manufacturer, is not guaranteed or endorsed by the publisher.

## Footnotes

1
http://greengenes.lbl.gov/Download/

## Supplementary Material

Supplementary Figure 1 Sequencing data. **(A)** Quality sequence length distribution map. **(B)** OTU rarefaction curves of intestinal flora in four groups of stool samples.
