# Supplementary figures and images for: Dysbiosis of the Gut Microbiome Is Associated With Histopathology of Lung Cancer
Source: Front Microbiol. 2022 Jun 14;13:918823. doi: 10.3389/fmicb.2022.918823 (PMC9237568; doi:10.3389/fmicb.2022.918823)

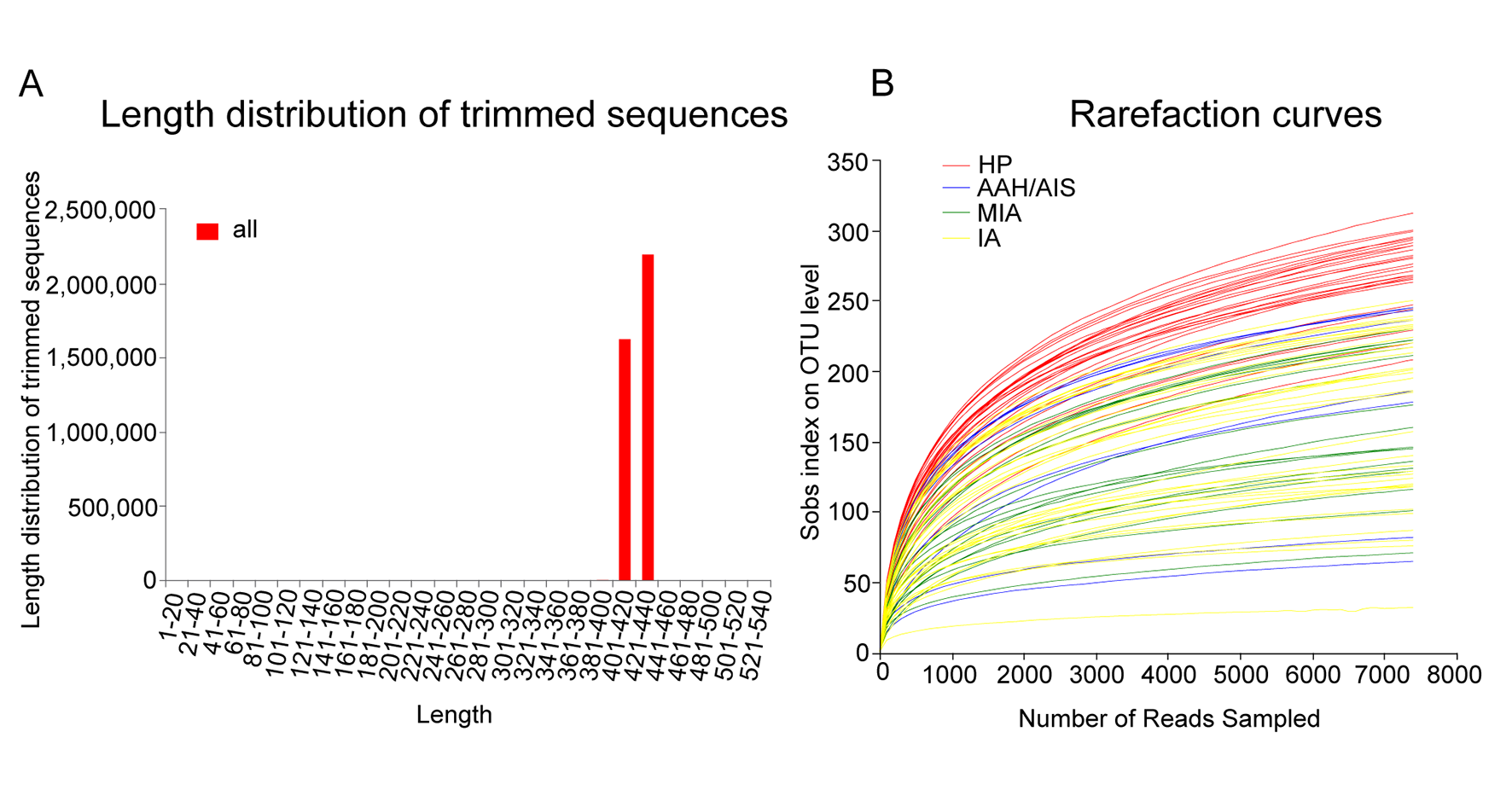

Supplement: Supplementary Figure 1 — Sequencing data. (A) Quality sequence length distribution map. (B) OTU rarefaction curves of intestinal flora in four groups of stool samples. [file Image_1.TIF]
