# Supplementary material for: Dysbiosis of the Gut Microbiome Is Associated With Histopathology of Lung Cancer
Source: Front Microbiol. 2022 Jun 14;13:918823. doi: 10.3389/fmicb.2022.918823 (PMC9237568; doi:10.3389/fmicb.2022.918823)
Supplement: Supplementary file 2 [file Table_1.DOCX]

**Table S1.** The topological features of correlation network

| Group | No. of nodes | No. of edges | Average degree | Average weighted degree | Average path length | Density | Average Clustering Coefficient |
| --- | --- | --- | --- | --- | --- | --- | --- |
| HP | 179 | 839 | 9.374 | 8.557 | 3.906 | 0.053 | 0.69 |
| AAH/AIS | 198 | 1266 | 12.788 | 15.872 | 3.919 | 0.065 | 0.79 |
| MIA | 224 | 1493 | 13.33 | 10.275 | 3.369 | 0.06 | 0.582 |
| IA | 229 | 1218 | 10.638 | 13.513 | 5.184 | 0.047 | 0.68 |
